# Health shock, medical insurance and financial asset allocation: evidence from CHFS in China

**DOI:** 10.1186/s13561-022-00400-z

**Published:** 2022-10-21

**Authors:** Yaxuan Liu, Yu Hao, Zhi-Nan Lu

**Affiliations:** 1 School of Management and Economics, Beijing Institute of Technology, 5 Zhongguancun South Street, Haidian District, 100081 Beijing, China; 2 School of Government, Peking University, 100871 Beijing, China; 3 Department of Government, London School of Economics and Political Science, WC2A 2AE London, UK; 4 Center for Energy and Environmental Policy Research, Beijing Institute of Technology, 100081 Beijing, China; 5 Beijing Key Lab of Energy Economics and Environmental Management, 100081 Beijing, China; 6 Sustainable Development Research Institute for Economy and Society of Beijing, 100081 Beijing, China; 7 Yangtze Delta Region Academy of Beijing Institute of Technology, 314001 Jiaxing, China; 8 Interventional Center of Valvular Heart disease, Beijing Anzhen Hospital, Capital Medical University, 100029 Beijing, China

**Keywords:** Health shock, Medical insurance, Financial asset allocation, Financial asset mobility, China

## Abstract

**Background:**

As health care cost is taking an increasingly substantial proportion of national wealth, health shocks and the subsequent medical expenditures have become increasingly vital contributions to financial risks. However, the individual or combined effects of social and financial medical insurance on household financial behaviors are poorly understood. This research aims to examine the effect of health shocks on financial asset mobility and portfolio allocation of the household. Also, whether medical insurance positively affects the financial market will be analyzed.

**Methods:**

Linear-regression models are used to determine the relationship between health shock, medical insurance, and household financial behaviors, including liquidity measures and financial portfolio (risk and risk-free assets). Two types of variables (transition probability and upward mobility) are constructed to measure the aggregate-level financial asset mobility. The portfolio of financial assets is categorized according to the risk it bears.

**Results:**

Households which experience health shocks are found to exhibit lower transition probability and upward mobility of financial assets than households that do not, and health shocks pose a more serious threat to low-income households. From the inter-temporal perspective, households that have medical insurance exhibit a higher probability of raising their position within the national financial asset distribution, and are more inclined to invest in the risky financial assets. Commercial insurance displays a larger marginal effect on financial asset allocation than social insurance. Our study results highlight an essential link between health shocks, medical insurance, and household financial behavior.

**Conclusion:**

This work identified and described the relationship between health-related factors (health shock and two types of medical insurance) and household financial behaviors (risky investment involvement and class mobility in financial asset). A strong link exists between the health and financial market, with heterogenous effects between urban and rural groups, households with distinct income levels, etc. A multilayered insurance system would be helpful to facilitate household income, financial consumption, and economic growth.

**Supplementary Information:**

The online version contains supplementary material available at 10.1186/s13561-022-00400-z.

## JEL

I31; I38; G52.

## Introduction

On a global scale, the financial market is booming, and national wealth is accumulating. For the latest summarization in 2021, there are 250 market infrastructure providers and 109 trillion dollars of equity market capitalization. As increasing significance is attached to financial products and services, residents’ concept of asset management is gradually changing. However, many individuals still hold conservative attitudes towards portfolio allocation, especially in developing countries. A comprehensive understanding of the factors that influence household portfolio allocation helps optimize relevant policies and satisfy the diversified needs of residents.

As medical costs take an increasing proportion of national income, health shocks and subsequent medical expenditures have become increasingly vital contributions to financial risks. Taking China as an example, the average life expectancy was estimated to be 77 years in 2018, while the number of years for healthy life expectancy was supposed to be 68.7 years^1^. With the acceleration of aging, the aggravation of workload, and pressure, the health crisis may affect individuals of all ages. In terms of medical expenses, according to the research of Mercer Consulting in 2019, On the global scale, the inflation rate of medical expenses is 9.6%, which is almost three times the current inflation rate (3.3%). In China, the gap is even extended (10.2% and 2.4%, respectively). The inflation rate of medical expenses in many other countries is more significant, like Malaysia (13.6%), the Philippines (13.7%), etc. Besides, the self-paid percentage of medical expenses in China is 16% in 2018. There is still a far way to go to reduce the medical burden on citizens, especially the poor. More than 42% of registered poor households in China return to poverty due to illness. The price of many services is climbing, including outpatient service, medicine, surgery, hospitalization, etc. There are many contributing factors to this problem. The demand for high-quality medical service far exceeds that of the supply of healthcare providers. Also, as the cost of advanced hospital infrastructure keeps increasing so as the salaries paid to the healthcare providers; notably, substantial medical expenses may exhaust life-long savings.

Medical insurance is intended to compensate residents for economic loss through the lateral spread of medical expenses. Government-led social insurance arrangements act as buffers for those who experience health shocks, but individuals are still exposed to the risk of extensive out-of-pocket (OCP)medical expenditure which could not be fully covered by social insurance. Many people want to have supplementary insurance no matter in employer-paid or self-enrolled way, in order to build an enhanced protection net. Quantifying the effects of each type of medical insurance is essential for ascertaining their role in the financial market. A detailed clarification of the differences between social and commercial medical insurance is presented in Table A3 in the appendix.

Although significant improvements have been made in the medical system reform, the urban-rural gap in medical insurance levels is still significant. Indeed, there is room for improvement in the fairness of the process and outcome of medical treatment. Table A4 in the appendix describes different medical insurance designs for rural and urban residents. The New Rural Co-operative Medical System’s actual reimbursement ratio is 46.4% in China, substantially below the reimbursement ratio in the Basic Medical Insurance System for Urban Workers and Residents, ranging from 60 to 70% on average in 2018. Moreover, unfairness exists among different income groups as well. The reimbursement amount for high-income individuals is significantly higher than for insured individuals with low income. The fragmentation of the social medical insurance system and the low coverage rate of the commercial medical insurance system both signal that financial behaviors are still largely influenced by health status.

This research examines the effect of health shocks and medical insurance on financial asset mobility and portfolio allocation. Does financial asset mobility vary with urban/rural areas, income level, or other characteristics? What are health shocks and medical insurance effects on financial asset mobility? Will medical insurance affect a household’s allocation of risky and risk-free financial assets? Are there any differences between commercial and social medical insurance, and how are they related? Does the urban-rural dual structure have significant impacts on the relationship of interest? Answers to these questions are critical for identifying the linkage between medical insurance and the capital market, which is crucial to reforming the medical insurance system.

This study contributes both to the literature and real practice. First, it contributes to the literature on the relationship between health and financial markets. We empirically analyze the impact of health shocks and medical insurance on household financial asset position mobility and portfolio allocation. Existing literature has concluded that health risks negatively impact household venture asset investment [e.g., 26, 16, 17]. However, to what extent medical insurance exerts mitigating effects on such negative relationship is still not well studied, especially in developing countries.[41]

Second, although there is a large amount of existing studies on households’ limited participation in financial consumption and investment [e.g., 18], few previous studies focus on the intermediary effect of risk preference in portfolio management from the perspective of social and commercial medical insurances. This research draws conclusions and proposes policy recommendations with profound practical significance and solid empirical evidence.

Third, this study is an important complement to the research on income mobility [e.g., 29] by focusing on financial market involvement. We provide empirical evidence of the effect of health risks and medical insurance on two aggregate-level financial asset mobility measures.

Fourth, even though previous scholars have studied the effect of private health insurance (PHI), only a few paid attention to the effect of supplemental private health insurance in the US [18]. There is limited empirical evidence on the combined effect of compulsory type (social insurance) and optional type (commercial insurance) in developing countries, where medical expenditure risk is still a critical drag on household financial decisions. We examined the comparative advantage, impact strength, and coordinated effect of two types of medical insurance (i.e., social insurance and commercial insurance). Our results indicate that the policy maker and practitioners should promote the diversified design of the medical insurance system without losing fairness and justice.

Last but not least, compared to previous studies, our research considers more comprehensive measures of financial behaviors in the form of absolute value (how much he/she invests in risky financial assets), proportion (what is the proportion of risky financial assets within the portfolio), and mobility (what is the upward movement probability of position within the national financial asset distribution). Additionally, we implement a heterogeneity analysis regarding the residential type (rural/urban), income level, etc.

The rest of the paper is organized in the following way. Section 2 reviews the related literature. Section 3 describes the data source and sample construction process, and presents the definitions of key variables. Section 4 presents our empirical framework, in which we introduce our identification strategy and econometric model. Section 5 shows the main empirical results and further estimation results on the interaction among different medical insurances, and the relationship between insurance premium investment and financial participations. Finally, Sect. 6 concludes and proposes policy suggestions.

## Literature overview

Why is a risky financial asset not widely holden by households? This question is referred to as the equity allocation (or stock-holding) puzzle, a vital topic in portfolio analysis [25, 7]. In the early stage, classical investment theory indicated that whether or not and to what extent to invest in risky assets depends on people’s risk attitude [32, 37]. With time going on, many other factors, such as health, housing, educational background, and age, were also gradually viewed as significant indicators. Income risk discourages risky investment because precautionary saving often rises when income risk goes up [23, 10, 31, 21, 6]. Households with elderly members are more inclined to hold stocks rather than tax-free bonds [34, 12] discussed the effect of housing on risky asset management and pointed out that housing prices crowd out investors’ shares in the stock market. [22] used the IV (instrumental variable) method and found that the IQ scores of investors are positively related to the probability of holding stocks. Many dimensions of explanation are proposed to answer this question, including trust, cost, and preference [24]. Other studies concentrate on alternative factors, such as human capital [5], enterprise assets [39], and insurance [20].

Health has become an increasingly pivotal part of human capital. As medical cost continues to be an essential contributor to financial risk [18], medical expenditure risk is seen as a background risk since it is not comprehensively insurable. Individuals who are exposed to background risk are hesitant to shoulder other risks, like risks in portfolio management. Theorists have alternately formalized this action as proper risk aversion [35], standard risk aversion [28], and risk vulnerability [19].

Health status has been regarded as a significant measure of background risk in many studies. [36] advocated that elderly individuals who think they are high-risk groups make a relatively conserved portfolio plan. [3] noted that poor health indirectly affects portfolio allocations by shrinking financial wealth. [15] found that individuals who believe that they would have to pay a large number of medical expenses in the next five years are inclined to hold financial assets with a lower share of risky types. Panel methods were further used in later studies, concluding that health exerts either slight or no effect on portfolio decisions [8, 13, 16, 30].

The huge burden of medical expenditures can be mitigated by medical insurance. In other words, insurance coverage affects the risk-bearing preference, both the income and substitution effects have been proposed in previous studies. In terms of the substitution effect, [27] found that social insurance could reduce the uncertainty risk and reduce preventive savings. In terms of the income effect, medical insurance in the form of insurance premiums reduces the disposal income of households, thus decreasing their risk investment [40, 9, 38, 8] proposed that with a universal medicare system, health status has no or very little significant influence on risky asset holdings among the retired based on research in Australia. [1] used the Survey of Health, Ageing, and Retirement in Europe to demonstrate that health risk affects portfolio choices only in countries with immature protective medicare systems. However, in developing countries, the effect of health insurance may not be as profound as that in developed countries. Therefore, this question needs further investigation in developing countries.

The literature related to financial asset allocation is gradually becoming enriched in developing countries. However, few studies have examined the contributors to portfolio choice from the perspective and health shock and insurance, let alone differentiating the distinct effects of social and commercial insurance types.

The compensation and risk incentive effects of insurance cannot be split either. In developing countries, the medical security system’s fairness and the financial market’s maturity are still in great need of improvement. This research addresses a critical policy issue: how do health shocks and medical insurance affect the mobility and allocation of households’ portfolios?

As medical expenditure continues to take a much higher substantial fraction of household resources, the portfolio allocation choice becomes to be largely influenced by health status. Households with less disposable income tend to invest less in risky financial assets, and their flatter wealth accumulation profile is likely to enlarge the income discrepancy. In consideration of the urban-rural dual structure in China, this study aims to provide a new perspective for facilitating financial consumption and economic growth.

## Data and variable construction

### Data source

The primary data source is two waves of the Chinese Household Finance Survey (CHFS) from 2015 to 2017. The CHFS was implemented by the Southwestern University of Finance & Economics and used a multistage, random cluster process to draw a sample of about 40,000 households in 29 provinces across China. Moreover, this survey is a nationally representative, biannual longitudinal survey of Chinese communities, families, and individuals. It covers household- and individual-level information, including housing, financial assets, debts, income, insurance, employment, demographic features, etc. The household- and individual-level data from 2015 to 2017 are combined according to household identification code. Since the head of a household mainly decides financial asset allocation, this study mainly focuses on the characteristics of the head and excludes the samples in which the head is not between ages 16 and 65. To solve the problem of missing data, the CHFS center has carried out interpolation processing on many variables. The interpolated data of the center used in our study undergoes truncation processing according to aggregate-level income. The household samples without any financial assets in the database are excluded because they do not have to decide on portfolio allocation if they possess none. In addition, samples with missing key variables or apparent anomalies were also excluded.

### Variables

#### Health shock

This article defines health shock as hospital admission in the year before the examination on financial behaviors. The CHFS asks the question, “Have you been hospitalized last year?”. If the respondent reports “yes”, a health shock is recorded between waves *t* and *t* + 1. There are several reasons that other definitions of health shock were not adopted. In general, we are interested in the effect of a dramatic health shock on individuals or their family members. Hospitalization is a better assessment than self-reported health status because hospitalization is relatively more objective than self-reported health status and can reduce the bias due to mental status. Indeed, some types of hospitalization may not be a negative shock, like child delivery surgery. But we do not have detailed information on the cause of hospitalization in our dataset; thus, we rule out the female subsample who are likely to give birth to a child at their age to conduct a robustness check. The decline in self-reported health is not as strong as hospitalization in capturing exogenous “shocks” to health. However, we still use the decline in self-reported health as the key variable in the robustness check. Moreover, individuals may not assess their self-reported health according to a consistent standard for an extended period, exposing the estimation results to bias. Therefore, a dummy for hospital admission was used in our study to examine the linkage between the financial market and health-related shock or investment.

Intuitively, health shock increases the household’s financial burden and prevents patients’ labor participation. These adverse effects are magnified when a health shock occurs to the household head. Therefore, we construct the health shock variable towards the household head and other family members separately.

#### Medical insurance

Both social and commercial medical insurance are investigated in this study. If residents possess at least one type of social medical insurance^2^, the indicator for social insurance would be recorded as 1. Otherwise, it is set as 0. In the survey sample, the coverage rate of social insurance is 93.9%. The coverage of social medical insurance is not 100% does not imply that it is not compulsory. In fact, those who are missing from the insurance coverage are randomly distributed in the population for non-systematic reasons.

Moreover, individuals or employers can purchase commercial medical insurance according to their willingness. Commercial medical insurance’s reimbursement amount and scope are more extensive than social medical insurance, and can also reimburse some medical expenditures that are not covered by social medical insurance. Therefore, commercial insurance not only reduces the burden of medical costs but also promotes the treatment process. Our dataset’s commercial medical insurance coverage rate is only 5.47%, which is far lower than that of social insurance.

#### Financial asset allocation

Household financial assets can be divided into risk-free and risky types. Risky financial assets include stocks, funds, bonds, financial derivatives (e.g., futures and warrants), and other financial products^3^ (e.g., bank financial products). Risk-free financial assets include cash, time deposits and demand deposits. In addition to financial assets, there are also productive assets, real estate, and so forth. Financial asset mobility and portfolio allocation are analyzed in this study. The probability of risky financial asset investment is either 0 or 1, and the proportion ranges from 0 to 1. Table 1 presents the holding rate and average value of various financial assets. It is true that insurance indeed belongs to financial assets, but medical insurance is solely used for health consumption. The liquidity of medical insurance is extremely low since it cannot be traded between the insured; therefore, the endogeneity problem is not severe. Households maximize lifetime utility within a multiperiod model, in which risky financial assets lead to higher expected returns and undergo higher volatility. Medical insurance can smooth the fluctuation of utility caused by health risks.

**Table 1:** Different Types of Household Financial Assets

Financial Asset	Full Sample		Urban Sample		Rural Sample	
	Holding Rate (%)	Mean (10,000 CNY)	Holding Rate (%)	Mean (10,000 CNY)	Holding Rate (%)	Mean (10,000 CNY)
Risk-free Kinds	99.81	5.87	99.70	8.10	99.97	2.81
Cash	96.21	0.79	96.33	0.97	96.05	0.51
Demand Deposit	68.95	4.42	73.57	5.71	62.59	2.34
Time Deposit	17.44	1.18	21.12	13.85	12.37	6.84
Risky Kinds	11.68	19.47	19.00	20.04	1.61	10.14
Stocks	6.70	13.75	11.12	13.97	0.62	8.30
Funds	3.48	10.10	5.74	10.34	0.38	5.07
Bonds	0.42	12.61	0.67	13.52	0.09	3.46
Financial products	4.43	21.24	7.10	21.95	0.75	11.97
Financial Derivative	0.06	9.13	0.10	9.13	0.00	0.00

Notes: The mean of market value is averaged according to the samples whose holding value is greater than zero

The holding rate and proportion of risky financial assets are presented in Table 2. It can be seen that households with medical insurance exhibit a higher probability of investing in risky financial assets than those not having medical insurance, whose holding rate (5.26%) and proportion (2.69%) of risky financial assets are very low.

**Table 2:** Holding Rate and Proportion of Risky Financial Assets

Households	Risky Financial Asset	
	Holding rate (%)	Proportion (%)
Only Commercial Insurance	18.78	10.95
Only Social Insurance	10.37	5.48
Both Commercial & Social Insurance	33.00	17.45
Neither Commercial nor Social Insurance	5.26	2.69

#### Individual and household controls

In the regression settings, both individual- and household-level characteristics are controlled in the regression to weaken the endogenous problem caused by omitted variables. Individual characteristics include gender, age, education level, marriage status, urban/rural status, and so forth. Household characteristics include income per family member, debt, urban or rural status, and family size. We also control for province-fixed effects in our further analysis. Descriptive statistics of the main variables are presented in Table 3.

**Table 3:** Descriptive Statistics

Variable	Full Sample	Sample w/ Health Shock	Sample w/o Health Shock	Diff (t-test)
Age	56.11	63.06	54.86	8.20***
	(13.46)	(12.85)	(13.18)	
Health status (= 1 if healthy, = 0 if unhealthy)	0.80	0.56	0.84	-0.28***
	(0.40)	(0.50)	(0.37)	
High school or above	0.31	0.25	0.32	-0.08***
	(0.46)	(0.43)	(0.47)	
Married	0.86	0.81	0.87	-0.06
	(0.39)	(0.39)	(0.33)	
Male	0.81	0.77	0.82	-0.05***
	(0.34)	(0.42)	(0.38)	
Annual income per member (10,000 CNY)	2.01	1.64	2.08	-0.45***
	(4.434)	(4.09)	(4.50)	
Family number	4.29	4.01	4.34	-0.33***
	(1.59)	(1.64)	(1.58)	
Rural	0.07	0.06	0.07	-0.01**
	(0.25)	(0.24)	(0.26)	
Observations	26,824	4,107	22,717	26,824

Notes: Standard deviations are in parentheses. “w/” stands for “with.” “w/o” stands for without. *** p < 0.01, ** p < 0.05, * p < 0.1

## Empirical framework

### Sample matching

There may be significant differences between the households with or without health shocks. A direct comparison between the two groups is not appropriate. Therefore, to reduce the selection bias, we apply the propensity score matching method (PSM) in estimating treatment effects based on observable characteristics. The propensity score is estimated based on a logistic regression model, in which the exposure to treatment (i.e., health shock) is used as dependent variable. Then propensity score is used to match treatment and control groups. We apply the method of covariate adjustment using the propensity score, using the covariates like age, gender, marriage status, residential type, education level, work status, health status, family size, etc. To estimate the treatment effect and its statistical significance, we much take the matched design into consideration [2]. For each treated individual, four “placebo-treated individuals” who have similar demographic and household characteristics but did not truly receive a health shock are matched to the treated individuals. This allows us to have a preliminary understanding of the relationship between household and financial behaviors^4^. Tables A1 and A2 in the appendix report the transition probability and upward mobility between *t*_0_ and *t*_1_ for samples with or without health shocks, respectively.

### Aggregate-level financial-asset mobility measures

In this sub-section, two financial asset mobility measures are constructed to quantify a household’s financial-asset mobility in the national financial-asset distribution, which is called aggregate-level financial-asset mobility.

#### Transition probability

Following [4], an intertemporal version of transition probability is proposed to measure the probability that a household is above the threshold *s* at time *t*_1_, conditioning on that the probability lies in below the threshold *s* at time *t*_0_. Let *F*_0_(·) and *F*_1_(·) denote the cumulative distribution function (c.d.f.) of the overall financial asset distribution at *t*_0_ and *t*_1_, respectively. The transition probability θ(*s*) at threshold *s* is represented as:

1 $$\theta (s)=\frac{{Pr\left[ {{F_1}\left( {{Y_1}} \right)>s,{F_0}\left( {{Y_0}} \right) \leqslant s} \right]}}{{Pr\left[ {{F_0}\left( {{Y_0}} \right) \leqslant s} \right]}}$$

This formula refers to the probability of a household ending up in a position higher than threshold *s* and is conditional on the household starting at a position lower than or equal to the threshold *s*. For instance, when *s* = 20%, it measures the bottom 20% of households by financial asset in *t*_0_ leave the lowest asset group in *t*_1_ which can be seen as “climbing out of the bottom financial asset trap”.

#### Upward mobility

Following [4] and [11], an intertemporal version of the upward mobility of financial assets is proposed based on the household’s position in the national financial asset distribution. This indicator quantifies the probability that a household’s financial asset position at time *t*_1_ exceeds its relative position at time *t*_0_ by a fixed amount τ. Let *F*_0_(·) and *F*_1_(·) denote the c.d.f. of the overall financial asset distribution at times *t*_0_ and *t*_1_. The upward mobility for a household that lies between thresholds *s*_1_ and *s*_2_ of *F*_0_(·) is denoted by:

2 $$\nu \left( {\tau ,{s_1},{s_2}} \right) = Pr\left[ \begin{array}{l}{F_1}\left( {{Y_1}} \right) - {F_0}\left( {{Y_0}} \right) > \\\tau \left| {{s_1} < {F_0}\left( {{Y_0}} \right) \le } \right.{s_2}\end{array} \right]$$

where *τ* governs the distance of the movement in the income distribution.

This paper considers the specific situation where $\tau =0$ to capture the growing tendency. Therefore, upward mobility is simplified as:

3 $$\nu \left( {{s_1},{s_2}} \right) = {\rm{Pr}}\left[ \begin{array}{l}{F_1}\left( {{Y_1}} \right) - {F_0}\left( {{Y_0}} \right) > \\0\left| {{s_1} < {F_0}\left( {{Y_0}} \right) \le } \right.{s_2}\end{array} \right]$$

Transition probability and upward mobility describe aggregate-level financial asset mobility from distinct perspectives. The transition probability is an absolute opportunity measure of financial market participation, i.e., the success rate of achieving a certain goal within the financial market. However, the absolute measure cannot reflect the initial position of a household, which exerts a greater effect on the intertemporal distribution changes. We might expect that a household that is occurred by health shocks is inclined to take money out of financial assets to cover medical expenditures. We may also expect that medical insurance can serve as a buffer layer to smooth health factors’ influence on financial behaviors. Upward mobility is constructed to measure the relative movement of a household’s financial assets within the overall distribution. It captures a household’s power to move upward from its original distribution of the financial asset. Upward mobility can capture the change in position that may not exceed a given threshold *s*. In summary, transition probability highlights the probability of reaching a certain threshold, while upward mobility emphasizes the household’s upward tendency relative to its original position.

For preliminary analysis, the effect of health shock occurrence on household financial asset mobility can be assessed by comparing the outcomes of households exposed to health shock. Then we apply the econometrics model to further investigate the relationship.

### Regression model

We apply LPM and OLS models to examine the effect of health shock and medical insurance on financial behaviors (Eq. (4)). National financial asset distribution is divided into five intervals: 0–20%, 20–40%, 40-60%, 60-80%, 80-100%. Each household locates in one of the intervals according to the position of its financial asset amount within the national distribution. The regression model is set as follows:

4 $$\begin{gathered} {Y_{i,hj}}=\alpha +{\beta _1}h{s_{i,hj}}+{\beta _2}hs{\__{i,hj}}+{\gamma _1}so{c_{i,hj}} \\ +{\gamma _2}com{m_{i,hj}}+{\delta _1}{Z_h}+{\delta _2}{X_i}+{\varphi _j}+{\varepsilon _{ihj}} \\ \end{gathered}$$

where *Y*_*i,hj*_ denotes the outcomes of household *h*, whose head is individual *i* in province *j*. One dimension of *Y*_*i,hj*_ is mobility measures, including transition probability and upward mobility. The other dimension of outcomes depicts their risk financial asset allocation, including a dummy for holding a risky financial asset or not, as well as a variable ranging from 0 to 1 that measures the proportion of risky financial assets. *hs*_*i,hj*_ is the indicator of health shock occurring to the head *i* between *t*_0_ and *t*_1_. Similarly, *hs_*_*i,hj*_ represents the health shock occurrence of the other family members of head *i* within household *h*, which equals 1 if health shock occurs, otherwise 0. We include two types of medical insurance in our model: *soc*_*i,hj*_ denotes social medical insurance and *comm*_*i,hj*_ denotes commercial medical insurance. Moreover, *X*_*i*_ is a set of individual controls of household head *i*, including gender, age and its square, marriage status, and education level. *Z*_*h*_ is a set of household-level controls, including family size, residential type (rural or urban) and income per family member. Our model permits the outcomes to vary among households in different provinces by adding province-fixed effects *φ*_j_. *ε*_*ihj*_ is the error term.

The coefficients *β*_1_ and *β*_2_ are the coefficients of interest, as they measure the effects of health shock and medical insurance on financial positions and behaviors. If health shock plays an essential role in household financial market participation, then *β*_1_ would be significantly different from zero. If there are significant differences between the mobility measures and portfolio allocation of households with or without medical insurance, *β*_2_ would be significantly different from zero. We also conducted a heterogeneity analysis to determine the distinct effects among different sample groups, providing more elaborate policy implications.

Higher returns usually accompany higher risk. In general, the market value of risky financial assets is much more volatile than that of risk-free financial assets. Therefore, risky financial asset is a crucial component that contributes to mobility. Uncertainty of health status adds to the risk of the financial market. Medical insurance, on one hand, has a direct wealth compensation effect on patients by reducing their financial burden. On the other hand, medical insurance also has a risk incentive effect, weakening the conservatism of households. In other words, medical insurance reduces precautionary savings and promotes financial consumption. Coefficients γ_1_ and γ_2_ are expected to be positive, meaning that having medical insurance is positively related to the relative position of household financial assets within the population.

## Empirical results and discussion

In this section, empirical results about the effects of health shock and medical insurance on household financial asset mobility and allocation are presented in detail. Possible explanations for why health shock and medical insurance matter are also proposed. We begin by analyzing the effects on household transition probability and upward mobility. Next, we examine how health shocks and medical insurance affect a household’s financial asset allocation. In addition, we explore whether health shocks and medical insurance have different economic implications for different subgroups by performing heterogeneity analysis along several dimensions.

### Effects on aggregate-level financial asset mobility

We first examine the effects of health shock and medical insurance upon aggregate-level financial asset mobility. Tables 4 and 5 present the estimation results, suggesting that health shocks, especially those that occur to the household head, decrease the transition probability and upward mobility of financial assets, while medical insurance leads to a substantial amount of mobility enhancement. The higher the threshold is, the more difficult it is to exceed. The estimation results also indicate that a health shock, either on the household head or on other family members, exhibits a significantly negative effect on transition probability, i.e., decreases the probability that a household ends up at a position higher than each threshold quantile. Health shocks occuring to the household head have a greater influence, in both economic and statistical significance, on the transition probability compared with health shocks occuring to other family members.

Table 5 presents the relationship among upward mobility^5^, health shock, and medical insurance. Health shocks exerts negative effects on upward mobility, though not in every group, meaning that the households under health shock are unlikely to place more capital in financial assets. The coefficients of medical insurance are mainly significant among those below the 40th percentile. In other words, for households with fewer financial assets, the medical insurance can exert more encouraging effects on them, compared with those who are rich in financial assets.

**Table 4:** Effects of Health Shocks and Medical Insurance on Transition Probability

	Transition Probability			
	Threshold: s = 20%	Threshold: s = 40%	Threshold: s = 60%	Threshold: s = 80%
	(1)	(2)	(3)	(4)
Health shock	-0.057***	-0.044***	-0.038***	-0.010*
	(0.015)	(0.011)	(0.008)	(0.005)
Health shock on family member	-0.023	-0.040***	-0.016**	0.003
	(0.015)	(0.011)	(0.008)	(0.005)
Social medical insurance	0.090***	0.074***	0.036***	0.015**
	(0.020)	(0.015)	(0.011)	(0.007)
Commercial medical insurance	0.076**	0.121***	0.097***	0.032**
	(0.032)	(0.028)	(0.020)	(0.013)
Individual controls	Yes	Yes	Yes	Yes
Household controls	Yes	Yes	Yes	Yes
Province FE	Yes	Yes	Yes	Yes
Observations	7341	11,232	16,453	21,751
R-squared	0.145	0.154	0.148	0.131

Notes: Standard errors are presented in parentheses. Individual control and family variables include gender, age, education level, marriage status, urban/rural status, income per family member, family size, and so forth. *** p < 0.01, ** p < 0.05, * p < 0.1

**Table 5:** Effects of Health Shocks and Medical Insurance on Upward Mobility

	Upward Mobility			
	Bottom 20%	20-40%	40-60%	60-80%
	(1)	(2)	(3)	(4)
Health shock	-0.057***	-0.019	-0.053***	-0.001
	(0.015)	(0.021)	(0.018)	(0.016)
Health Shock on family member	-0.023	-0.054**	-0.004	0.011
	(0.015)	(0.021)	(0.017)	(0.016)
Social medical insurance	0.090***	0.109***	0.018	0.020
	(0.020)	(0.030)	(0.027)	(0.023)
Commercial medical insurance	0.076**	0.101**	0.050	0.018
	(0.032)	(0.040)	(0.031)	(0.026)
Individual controls	Yes	Yes	Yes	Yes
Household controls	Yes	Yes	Yes	Yes
Province FE	Yes	Yes	Yes	Yes
Observations	7341	3891	5221	5298
R-squared	0.145	0.120	0.118	0.131

Notes: Standard errors are presented in parentheses. Individual control and family variables include gender, age, education level, marriage status, urban/rural status, income per family member, family size, and so forth. *** p < 0.01, ** p < 0.05, * p < 0.1

### Effects on financial asset allocation

Tables 6 and 7 present the effect of medical insurance on the probability and proportion of holding risky financial assets. Health shocks hinder risky financial investment to some extent. Medical insurance could promote risk investment at the household level, while the marginal effect of social medical insurance is lower than that of commercial insurance. As shown in Table 6, the coefficient of commercial medical insurance is significant at 1% for all groups except those at the bottom 20th percentile. However, the coefficient of social medical insurance has a smaller magnitude and significance level, indicating the larger impact of commercial insurance on the risky investment tendency.

Besides, for those at the bottom 20th percentile, both social and commercial medical insurance’s coefficients are insignificant (as shown in column (1)). This means both commercial and social medical insurance fail to lift their inclination to invest in risky financial assets.

Moreover, the magnitude of the coefficients of medical insurance exhibits a growing tendency from low to high percentiles. Intuitively speaking, with the position climbing up, risk investment tendency is more likely to be boosted by medical insurance. For example, as shown in column (5) of Table 6, conditional on other variables unchanged, having commercial medical insurance increases the probability of holding risky financial assets by 10.3% for those whose financial assets are distributed in the top 20% group within the population. In contrast, for those distributed between 20 and 40%, the effect of commercial medical insurance shrinks to 7.1%. A similar pattern can be found in Table 7.

For those at the bottom 20th percentile, commercial and social medical insurance fail to lift their inclination of investment in risky financial assets. In the higher percentiles, medical insurance exerts a more significant effect in promoting risky financial asset allocation. Commercial insurance is still more profound in its impact on financial market participation than social insurance.

**Table 6:** Effects of Health Shocks and Medical Insurance on the Probability of Holding Risky Financial Assets

	Holding Risky Financial Asset				
	Bottom 20%	20-40%	40-60%	60-80%	Top 20%
	(1)	(2)	(3)	(4)	(5)
Commercial medical insurance	0.023	0.071***	0.073***	0.077***	0.103***
	(0.017)	(0.027)	(0.021)	(0.022)	(0.022)
Social medical insurance	0.002	0.002	0.021**	-0.016	0.085***
	(0.004)	(0.009)	(0.010)	(0.016)	(0.024)
Health Shock	-0.001	0.003	-0.010	0.023*	-0.001
	(0.003)	(0.006)	(0.007)	(0.012)	(0.020)
Health shock on family member	0.006	-0.004	0.012	0.004	-0.032*
	(0.004)	(0.005)	(0.008)	(0.011)	(0.018)
Individual controls	Yes	Yes	Yes	Yes	Yes
Household controls	Yes	Yes	Yes	Yes	Yes
Province FE	Yes	Yes	Yes	Yes	Yes
Observations	7341	3891	5221	5298	4825
R-squared	0.055	0.065	0.095	0.107	0.176

Notes: Standard errors are presented in parentheses. Individual control and family variables include gender, age, education level, marriage status, urban/rural status, income per family member, family size, and so forth. *** p < 0.01, ** p < 0.05, * p < 0.1

**Table 7:** Effects of Health Shocks and Medical Insurance on the Proportion of Holding Risky Financial Assets

	Proportion of Risky Financial Asset				
	Bottom 20%	20-40%	40-60%	60-80%	Top 20%
	(1)	(2)	(3)	(4)	(5)
Commercial medical insurance	0.016	0.039**	0.030**	0.038***	0.066***
	(0.011)	(0.017)	(0.012)	(0.014)	(0.015)
Social medical insurance	0.003	-0.003	0.011*	-0.018	0.044***
	(0.002)	(0.007)	(0.005)	(0.011)	(0.015)
Health shock	-0.001	0.000	-0.005	0.019**	-0.001
	(0.002)	(0.004)	(0.004)	(0.008)	(0.013)
Health shock on family member	0.002	-0.001	0.002	-0.000	-0.022*
	(0.002)	(0.003)	(0.004)	(0.006)	(0.012)
Individual controls	Yes	Yes	Yes	Yes	Yes
Household controls	Yes	Yes	Yes	Yes	Yes
Province FE	Yes	Yes	Yes	Yes	Yes
Observations	7341	3891	5221	5298	4825
R-squared	0.043	0.055	0.080	0.084	0.146

Notes: Standard errors are presented in parentheses. Individual control and family variables include gender, age, education level, marriage status, urban/rural status, income per family member, family size, and so forth. *** p < 0.01, ** p < 0.05, * p < 0.1

Table 8 presents the heterogeneity analysis on different income groups, which are divided by the inclination median income level. Health shock significantly decreases the probability and proportion of risky financial assets among the low-income group, meaning that low-income households are more likely to withdraw from the financial market once they are exposed to health risks. The risk incentive effects of commercial medical insurance work for both high- and low-income households, and the marginal effect is a little bit higher for the high-income group. Moreover, social medical insurance’s impact on risk investment is mainly exhibited in the high-income group. The insignificance of social medical insurance on low-income groups can be explained as follows. First of all, based on the fundamental realities of China, social medical insurance acts as a very basic protection cover for the poor. There are many types of the medical cost that cannot be fully covered by social medical insurance, including treatment in (1) non-designated hospital service (except for emergency treatment); (2) occupational disease, injury at work, or recurrence of industrial injury; (3) injuries caused by traffic accidents; (4) injury caused by violation of the law; (5) food poisoning caused by a responsible accident; (6) suicide treatment; (7) injuries caused by medical accidents; and medical expenses that shall be paid by themselves according to national and municipal regulations. In other words, even with social medical insurance, the poor are still at a high risk of being exposed to medical costs. The high-income group can better handle the heavy medical expenses, whether with or without commercial medical insurance. However, for the low-income group, social medical insurance cannot play such an essential role in influencing risky financial asset allocation. Besides, the coverage rate of social medical insurance is very high; thus, the variation of this independent variable is relatively low, which can result in a lower significance level of it compared to commercial medical insurance. This may be a result of the imperfect system design. More medical resources are allocated to high-income individuals, which reveals massive inequality among them.

**Table 8:** Heterogeneity Analysis among High-income and Low-income Households

	Holding Risky Financial Asset		Proportion of Risky Financial Asset	
	High-income	Low-income	High-income	Low-income
	(1)	(2)	(3)	(4)
Commercial medical insurance	0.111***	0.088***	0.069***	0.055***
	(0.020)	(0.029)	(0.013)	(0.021)
Social medical insurance	0.064***	0.008	0.034**	0.002
	(0.025)	(0.005)	(0.015)	(0.004)
Health shock	-0.004	-0.007***	0.001	-0.005***
	(0.019)	(0.002)	(0.013)	(0.002)
Health shock on family member	-0.011	-0.002	-0.013	-0.000
	(0.018)	(0.004)	(0.011)	(0.003)
Individual controls	Yes	Yes	Yes	Yes
Household controls	Yes	Yes	Yes	Yes
Province FE	Yes	Yes	Yes	Yes
*P value for Social MI*	*0.0266*		*0.0454*	
*P value for Commercial MI*	*0.5193*		*0.5624*	
Observations	5315	5315	5315	5315
R-squared	0.125	0.224	0.111	0.199

Notes: Standard errors are presented in parentheses. Individual control and family variables include gender, age, education level, marriage status, urban/rural status, income per family member, family size, and so forth. *** p < 0.01, ** p < 0.05, * p < 0.1

The urban-rural dual structure still stands out in China. Table 9 shows the distinct impacts of medical insurance on rural and urban households. After controlling for other variables, both social and commercial medical insurance encourage risk investment in households with urban hukou, while the marginal effect of commercial medical insurance is relatively larger than that of social medical insurance. Rural households’ risk investment incentive is stimulated by commercial insurance instead of social insurance, which also implies urban-rural inequality. In other words, investing in risky financial assets has little to do with medical insurance for residents with rural hukou. However, if the investment initiative of urban residents could not be motivated, the overall financial market would lose loads of potential participants.

The benefits of social medical insurance for rural residents are subjected to several criticisms, which can partially explain the insignificant effects of social medical insurance on financial decisions. First, rural residents exhibit a low satisfaction rate toward social medical insurance. Second, the low level of coverage by social medical insurance leads to little impact on the risk awareness of rural residents. Third, rural residents are still not fully aware of social medical insurance, which requires a cumbersome reimbursement procedure. Fourth, the insurance premium is rising every year. It has increased to 350 yuan per year per individual, which can be a financial burden for the poor.

**Table 9:** Heterogeneity Analysis among Rural and Urban Households

	Holding Risky Financial Asset		Proportion of Risky Financial Asset	
	Rural	Urban	Rural	Urban
	(1)	(2)	(3)	(4)
Commercial medical insurance	0.042***	0.124***	0.020***	0.071***
	(0.012)	(0.016)	(0.007)	(0.011)
Social medical insurance	0.003	0.034***	0.000	0.014*
	(0.004)	(0.012)	(0.003)	(0.008)
Health shock	0.004	-0.012	0.004**	-0.008
	(0.003)	(0.009)	(0.002)	(0.006)
Health shock on family member	0.005*	-0.017*	0.002	-0.013**
	(0.003)	(0.010)	(0.002)	(0.006)
Individual controls	Yes	Yes	Yes	Yes
Household controls	Yes	Yes	Yes	Yes
Province FE	Yes	Yes	Yes	Yes
Observations	15,313	11,263	15,313	11,263
R-squared	0.066	0.163	0.048	0.129

Notes: Standard errors are presented in parentheses. Individual control and family variables include gender, age, education level, marriage status, urban/rural status, income per family member, family size, and so forth. *** p < 0.01, ** p < 0.05, * p < 0.1

### Discussion

In fact, the mobility measures are related to the amount of investment, while the portfolio choice is related to cross-group allocation between risky and risk-free types. Whereas both health shock and medical insurance matter for the amount of investment, only the latter matters for risky asset allocation. This can be due to the various and inconsistent paths to change the asset allocation. According to your suggestion, the discussion is put into the conclusion section instead of the results.

We propose explanations for the insignificance of health shock in various pathways. On the one hand, health shock occurrence may decrease the risky financial asset allocation in reducing disposable income for other uses. When a health shock occurs to the household head or a family member, the treatment process can add a heavy burden of medical costs, leaving little savings for risky financial asset investment. On the other hand, if the family finds that the medical expenditure may be unaffordable, they may choose to invest in high-return assets for purpose of speculation. The household may hesitate to withdraw the investment in risky financial assets because it may cost much service charges and even default fees. In other words, health shock can also positively affect risky financial asset allocation. Due to the opposite effects mentioned above, the insignificance of health shock (or hospitalization) is not counter-intuitive. We also provide empirical evidence of the effects of health shock and medical insurance on detailed types of risky financial assets, including stock (Table A5 in the appendix), the fund (Table A6 in the appendix), bond/financial derivatives, or financial instruments (Table A7 in the appendix).

The financial consequences of illness are potentially far-reaching. The household may spend down their accumulated assets [14], including financial assets. There are other points of view that a reduction in health stock between adolescence and young adulthood is even more profoundly related to the willingness of risk-taking in later years [41]. This means that the relationship between risk attitude and health shock may exert heterogenous effects in different age groups, thus we cannot directly infer an exact pathway.

To better understand the logic from Tables 6, 7 and 8, we provide further evidence and explanations. Table 6 reveals the distinct effects of insurance on households with different amounts of asset holding, which can be seen as a measure of economic status. Table 8 further confirms such heterogeneous effects using household income as another measure of household economic status. Table 10 is closely related to them because *Log of Commercial Medical Insurance Premium* is positively related to investment amount (rich). Considering the condition of insurance premium in China, social medical insurance premium is decided by the administrator under a low level of marketization. At the same time, the average premium standard continuously increased during the past few years (as shown in Figure A2 in the appendix). According to a recent report by Oliver Wyman in 2022^6^, China’s life insurance premium is expected to surpass the US market and become the world’s largest market by 2030. Healthcare in China requires individuals to prepay for the services out of their own pocket^7^. Under such circumstances, the insurant may hesitate to invest in risky financial assets with the increase of social medical insurance. This explains the weakening effect of social medical insurance premiums upon risky financial investment.

To further validate your hypothesis that Log of Commercial Medical Insurance Premium is positively related to the investment amount (rich), we calculate the correlation coefficient between them: 0.09. This is intuitive in consideration of the voluntary investment in commercial medical insurance, whose premium payment would be in accordance with an individual’s willingness to pay.

On the other hand, log of social medical insurance premium relates oppositely to risky asset holdings in Table 10. This can be partially validated by the correlation coefficient between log of social medical insurance premium and risky financial asset holding: -0.04. The negative relationship could also be seen from the correlation figure in Figure A3 in the appendix. It is noteworthy that many Chinese citizens are not fully aware of the value of long-term, ongoing investment in social health insurance [33], especially when patients are still exposed to significant out-of-pocket sums with the coverage of social medical insurance.

### Robustness analysis

#### Impacts of other types of insurance

Apart from medical insurance, the social security system in China also includes social endowment insurance, social unemployment insurance, etc. Commercial insurance includes medical insurance and life insurance, and their impacts may interact with each other. Therefore, ignoring other insurance may lead to estimation errors. We also extend the attention from medical insurance to other insurance (as shown in Table A8 in the appendix). The result also shows the importance of social and medical insurance on the financial market even after including other types of insurance. The impact of commercial medical insurance is still significant, especially on urban households.

#### Alternative models

For robustness checks, other proxy variables are substituted for key variables in the model. The premium of medical insurance, which is a continuous variable, is used as the substitution variable for the dummy of medical insurance. The results are similar to baseline regression results, as depicted in Table A9 in the appendix. In addition, the logit regression model and linear probability model (LPM) regression model are also used for robustness check. The empirical result is presented in Table A10 in the appendix, leading to similar conclusions. Moreover, we run another regression model by replacing health shock with a decline in self-reported health status. We still obtain similar conclusions for three types of outcomes, as shown in appendix Tables A11, A12, A13 and A14, which further verify the robustness of our findings.

#### Instrument variable estimation

As social medical insurance systems worldwide are under compulsory enrolment, there is limited room for voluntary participation. However, commercial medical insurance is different because people can choose whether or not to buy it. Individuals who prefer risky financial assets may also have a greater tendency to purchase commercial medical insurance. In addition, commercial insurance insurers may also choose specific customers to pursue profits through unfair insurance. Commercial insurance insurers are willing to sell the insurance to healthy citizens with a lower risk of falling ill, so they are unlikely to shoulder high medical costs during the insurance period. However, for those diagnosed with acute or chronic diseases, the insurers may increase the insurance premium or even refuse the order to avoid medical expenditures. The adverse selection tendency of insurance issuers is contrary to the original intention of “serving as a buffering and protection net for individuals with diseases.” Therefore, the instrumental variable method is applied to check for the robustness of conclusions in the paper. The average commercial medical insurance purchase rate in the specific county is taken as the instrumental variable. The coverage of commercial medical insurance of households in the same county influences the insurance selection decision of the household itself. However, it would not directly affect the portfolio allocation. Tables A15, A16, A17, and A18 in the Appendix present the estimation results of IV regressions on the same research question as presented in Sect. 4. Similar findings are reached, which proves the robustness of our conclusions.

## Further analysis

We further analyzed the relationship between the medical insurance premium and financial asset allocation. As mentioned before, social medical insurance is enforced by the government in China. Individuals regularly pay a certain insurance premium, and obtain the option of being reimbursed if they are exposed to medical expenditures during the period covered by insurance. Either individuals or employers can purchase commercial insurance. Insurance premium can be regarded as a kind of preventive health investment. What is the relationship between the insurance premium and financial behaviors?

Table 10 reveals the estimation results of the effect of medical insurance on risky financial asset investment. Commercial medical insurance exhibits significantly positive coefficients both in columns (1) and (2), which can be seen as a “multiplier” effect. Traditionally, the multiplier effect occurs when investment in medical insurance causes a bigger final increase in risky financial assets. This is a borrowed term from the macroeconomics field. However, the increasing input into social medical insurance is negatively related to allocation in risky financial assets. This can be due to the compulsoriness of being involved in the social medical security system, especially when the insurance premium is rising nearly every year and becomes an increasing financial burden for those with little risk awareness towards health.

**Table 10:** Medical insurance and Risky Financial Asset Investment

	Holding Risky Financial Asset	Proportion of Risky Financial Asset
	(1)	(2)
Log of Commercial Medical Insurance Premium	0.013***	0.008***
	(0.002)	(0.001)
Log of Social Medical Insurance Premium	-0.002**	-0.002***
	(0.001)	(0.001)
Health shock	0.001	0.001
	(0.004)	(0.003)
Health shock on family member	-0.004	-0.004
	(0.004)	(0.002)
Individual controls	Yes	Yes
Household controls	Yes	Yes
Province FE	Yes	Yes
Observations	24,188	24,188
R-squared	0.193	0.155

Notes: Standard errors are presented in parentheses. Individual control and family variables include gender, age, education level, marriage status, urban/rural status, income per family member, family size, etc. *** p < 0.01, ** p < 0.05, * p < 0.1

## Conclusions and policy implications

China’s economy is under a high growth rate, including the financial market. Generally, financial asset investment exerts a significant effect on households’ disposable income. Financial asset mobility is an equalizer of long-term class inequality and a roll booster for income mobility. Therefore, understanding the key factors that help households increase financial asset mobility is of considerable significance.

The main findings of this article are listed as follows. First of all, in terms of mobility measures, health shock, either on the household head or other family members, hinders the upward changes of position within the national distribution. Medical insurance, especially commercial insurance, exerts a positive effect on the mobility of household financial asset position within the national distribution. Second, in terms of asset allocation choice, having medical insurance is positively related to risky financial investment, while health shock has an insignificant effect on financial asset allocation (reasons have been proposed in Sect. 5.3 discussion). Third, for heterogenous analysis, we conduct several sub-group analyses and find that low-income households are more likely to withdraw their risky financial asset and transform into risk-free asset types once they are exposed to health shocks. The risk incentive effects of commercial medical insurance work for both the high- and low-income households, with a little bit higher marginal effect on high-income groups. In other words, the effect of commercial medical insurance is larger for richer people (who have larger assets). At the same time, social medical insurance’s impact on risk investment mainly exhibits in the high-income group.

Our research contributes to the literature and policy-making process in the following perspectives. First, we find that medical insurance, especially the supplemental commercial type, can serve as a protection net by mitigating medical out-of-pocket payment risk and incentivize risky financial asset allocation. Medical insurance can be seen as a link between health inputs/outcomes and financial behaviors. Although previous studies have studied the effect of private health insurance (PHI), only a few paid attentions to the effect of supplemental private health insurance in the US [18]. There is limited empirical evidence on the combined effect of compulsory type (social medical insurance) and optional type (commercial medical insurance) in developing countries, where medical expenditure risk is still a critical drag of household financial decisions. There is another significant difference between the US market and the Chinese market. In the US, government-led health insurance includes Medicare, Medicaid, and the Children’s Health Insurance Program (CHIP), which are mainly focused on people over 65, the disabled, and children. Considering the market-oriented medical service system in the US, high medical costs are an extremely heavy burden for ordinary people. Therefore, commercial health insurance has become a “rigid need.“ But in China, the medical security system mainly consists of medical insurance for urban residents, medical insurance for urban workers, urban and rural medical assistance, and the new rural cooperative medical care system. Basic medical insurance for urban workers, basic medical insurance for urban residents and new rural cooperative medical care are the main parts of the medical insurance system. Commercial medical insurance and critical illness medical insurance are positioned as supplementary layers as presented in the figure below. There is no clear consensus on whether commercial insurance plays a similarly significant role in financial behaviors.

Besides, compared to previous studies, our research considers more comprehensive measures of financial behaviors, in the form of absolute value (how much he/she invests in risky financial assets), proportion (what is the proportion of risky financial assets among all), and mobility (what is the probability of upward movement within the population). Therefore, we can provide a rounded picture of the impact of medical insurance on financial asset allocation. Investigating the impact of medical insurance on risk attitude in the realm of financial asset allocation can provide evidence on how medical insurance mitigates the health shock’s effect on financial decisions.

Moreover, health shock does not exert a significant effect on risky financial asset allocation, though exerts a relatively significant effect on mobility measures.

The following policy suggestions are proposed based on research findings. First, optimizing commercial medical insurance design is of considerable significance in encouraging financial consumption. Commercial medical insurance plays a prominent role in promoting upward mobility, but the current purchase rate of commercial medical insurance is relatively low. Second, the rural-urban dual structure pattern is still prevalent in China. Few rural households invest in risky financial assets. Medical insurance for rural residents, such as New Rural Cooperative Medical Insurance, has achieved comprehensive coverage but a low level of security; thus, rural households are still exposed to a high risk of medical expenditures if health shocks occur. Establishing a unified insurance system is essential to narrow the urban-rural gap and reduce inequality. Third, social medical insurance should be refined to act as a social security net, especially protecting poverty from becoming stuck in the low-income trap caused by health shocks. If a multilayered insurance system could be constructed, households would be more likely to increase financial consumption, enhancing household income and facilitating economic growth from the macro perspective. The policy implications in China, the largest developing country in the world, can also apply to other countries (e.g., BRICS^8^).

## Electronic supplementary material

Below is the link to the electronic supplementary material.

Supplementary Material 1

## Data Availability

All data are available upon application.

## List of abbreviations

CHFS: Chinese Household Finance Survey
BRICS: Brazil, Russia, India, China, and South Africa
IV: instrumental variable

## Footnotes

1 Source: National Health Commission.

2 Social medical insurance include Social medical insurance includes Basic Medical Insurance for Urban Employees, Basic Medical Insurance for Urban Residents, New Rural Cooperative Medical Insurance, Basic Medical Insurance for Urban and Rural Residents, and Public Medical Insurance.

3 Financial products refer to those with a subscription starting point of more than 50,000 yuan, excluding the Internet financial products and P2P online lending and crowdfunding.

4 The matching result is shown in Figure A1 in the appendix.

5 The probability that a household ends up in a financial asset group higher than its original one, with an interval of 20%.

6 Source: Report: China’s Life Insurance Premium To Surpass The Us Market By 2030 And Reach 45 Trillion Yuan By 2050 (oliverwyman.com).

7 Source: Guide to Health Insurance and Healthcare System in China | InterNations.

8 BRICS includes Brazil, Russia, India, China and South Africa.
