# Supplementary material for: Health shock, medical insurance and financial asset allocation: evidence from CHFS in China
Source: Health Econ Rev. 2022 Oct 21;12:52. doi: 10.1186/s13561-022-00400-z (PMC9585822; doi:10.1186/s13561-022-00400-z)
Supplement: Supplementary file 1 — Supplementary Material 1 [file 13561_2022_400_MOESM1_ESM.docx]

**Appendix**

**Part A: comparison of mobility measures between sample with health shock vs. matched sample without health shock**

Table A1 reports the transition probability between period t_0_ (the wave before the health shock) and period t_1_ (the wave after the health shock) in our two samples, with and without health shock, respectively. The samples were matched according to characteristics before health shocks, including age, gender, education level, marriage status, self-reported health status, work status, rural or urban living status, family size, etc. Therefore, the transition probabilities of the groups with or without health shocks are comparable, and health shocks mainly cause the differences in mobility indicators between the two groups. The disparity in transition probabilities between the groups with or without health shocks directly measures the difference in the economic opportunities between them. Households that went through health shocks have a much lower transition probability than those that did not. Given that a household lies in the bottom quintile of the financial asset distribution in wave t_0_, the chance of transiting out of the bottom quintile in wave t_1_ is 60.5% if the household does not suffer from a health shock. However, the chance decreases by 5.8 percentage points to 54.7% if the household experiences a health shock. Similarly, households that lie in other quintiles are more likely to lift their position without health shocks.

Table A1: Transition Probability

| Percentile | W/O health shock | W/ health shock | Difference |
| --- | --- | --- | --- |
|  | (1) | (2) | (3) |
| 20% | 0.605 | 0.547 | 0.058*** |
|  | (0.489) | (0.498) | (0.014) |
| 40% | 0.369 | 0.305 | 0.064*** |
|  | (0.483) | (0.460) | (0.011) |
| 60% | 0.231 | 0.191 | 0.040*** |
|  | (0.422) | (0.393) | (0.008) |
| 80% | 0.096 | 0.090 | 0.005 |
|  | (0.294) | (0.286) | (0.005) |

Notes: The transition probability measures the probability that a household ends up at a position higher than the sth quantile in wave t, conditioning on starting at a position lower than or equal to the sth quantile in wave t − 1. For example, the row “80%” means the probability that a household ends up at a position higher than the 80th quantile in wave t, conditioning on starting at a position lower than or equal to the 80th quantile in wave *t – 1*. Health shock occurs between t_0_ and t_1_. “W/O” denotes “without,” and “W/” denotes “with.” The “W/O health shock” and “W/ health shock” columns report the transition probabilities for the households under different health conditions. Here we mainly focus on the health shock experienced by the household head. The “Difference” columns report the difference in the transition probabilities between the two groups. Standard errors are in parentheses.

Next, upward mobility is investigated from the perspective of another indicator. Households are divided into five subgroups based on their financial asset position before the health shock, each representing 20 percentile in the national financial asset distribution. Upward probabilities in five subgroups are presented in Table A2 separately. Take the 20%-40% group as an example for those that experience health shock(s), their probability of lifting their position to 40% or above is 6.4 percentage points lower than those without health shock(s).

Table A2: Upward mobility

| Financial Asset Group | W/O health shock | W/ health shock | Difference |
| --- | --- | --- | --- |
|  | (1) | (2) | (3) |
| Bottom 20% | 0.605 | 0.547 | 0.058*** |
|  | (0.489) | (0.498) | (0.014) |
| 20%-40% | 0.473 | 0.408 | 0.065*** |
|  | (0.499) | (0.492) | (0.002) |
| 40%-60% | 0.343 | 0.303 | 0.040** |
|  | (0.475) | (0.460) | (0.017) |
| 60%-80% | 0.200 | 0.221 | 0.020 |
|  | (0.400) | (0.415) | (0.014) |

Notes: Health shock takes place between t_0_ and t_1_. “W/O” denotes “without,” and “W/” denotes “with.” The “W/O health shock” and “W/ health shock” columns report the transition probabilities for the households under different health conditions. Here we mainly focus on the health shock experienced by the household head. The “Difference” column reports the difference in the transition probabilities between the two groups. Standard errors are in parentheses.

**Part B: Tables and figures for the complementary results**

Table A3: Differences Between Social and Commercial Medical Insurance

|  | Social medical insurance | Commercial medical insurance |
| --- | --- | --- |
| Orientation | Social welfare | Profit-oriented |
| Coverage of reimbursement | Nearly all kinds of large & little diseases | Specific deceases |
| Fund-raising source | Country, work unit, and individual | Insurer |
| Condition for insurance | N/A | Rules set by the insurance company |
| Main responsible party | Government | Insurance company |

Table A4: Different Types of Medical Insurances

| Type | Description |
| --- | --- |
| Basic Medical Insurance for Urban Employees | For urban workers (on-the-job personnel and retirees)  The worker himself bears insurance premiums, and part of it is undertaken by the employer  Normally this kind of health care is one of the highest proportions of reimbursement, corresponding insurance cost is higher, and also need to pay by the month. |
| Basic Medical Insurance for Urban Residents | For those that did not obtain employment in the city;  Voluntary by the individual; Insurance cost is at one's own expense, and the government also can give an allowance |
| New Rural Cooperative Medical Insurance | For rural residents (not all rural registered permanent residence);  Voluntary;  Insurance cost is at one's own expense, and the government also can give allowance;  Generally, there will be a pharmaceutical limited. Only the specified directory of drugs can submit an expense account;  The payment is for one year. |

Table A5: Effects of Hospitalization and Insurance on Stock Holding

|  | Holding Stock | | | | |
| --- | --- | --- | --- | --- | --- |
|  | Bottom 20% | 20%-40% | 40%-60% | 60%-80% | Top 20% |
|  | (1) | (2) | (3) | (4) | (5) |
| Commercial medical insurance | 0.022 | 0.024 | 0.047** | 0.062*** | 0.101*** |
|  | (0.017) | (0.020) | (0.019) | (0.021) | (0.021) |
| Social medical insurance | -0.004 | 0.010* | 0.014 | -0.019 | 0.038* |
|  | (0.005) | (0.006) | (0.010) | (0.016) | (0.023) |
| Health shock | -0.003 | -0.001 | 0.001 | 0.011 | -0.007 |
|  | (0.003) | (0.005) | (0.007) | (0.011) | (0.017) |
| Health shock on family member | 0.004 | -0.005 | 0.009 | -0.005 | -0.039** |
|  | (0.004) | (0.005) | (0.007) | (0.010) | (0.016) |
| Individual controls | Yes | Yes | Yes | Yes | Yes |
| Household controls | Yes | Yes | Yes | Yes | Yes |
| Province FE | Yes | Yes | Yes | Yes | Yes |
| Observations | 7341 | 3891 | 5221 | 5298 | 4825 |
| R-squared | 0.067 | 0.053 | 0.082 | 0.110 | 0.170 |

Notes: Standard errors are presented in parentheses. Individual control and family variables include gender, age, education level, marriage status, urban/rural status, income per family member, family size, and so forth. *** p<0.01, ** p<0.05, * p<0.1.

Table A6: Effects of Hospitalization and Insurance on Fund Holding

|  | Holding Fund | | | | |
| --- | --- | --- | --- | --- | --- |
|  | Bottom 20% | 20%-40% | 40%-60% | 60%-80% | Top 20% |
|  | (1) | (2) | (3) | (4) | (5) |
| Commercial medical insurance | 0.009 | 0.026 | 0.011 | 0.040*** | 0.064*** |
|  | (0.010) | (0.016) | (0.011) | (0.015) | (0.017) |
| Social medical insurance | 0.004*** | 0.003 | 0.006 | -0.012 | 0.002 |
|  | (0.001) | (0.005) | (0.005) | (0.010) | (0.017) |
| Health shock | 0.002 | 0.001 | 0.002 | 0.013* | -0.010 |
|  | (0.002) | (0.004) | (0.004) | (0.007) | (0.013) |
| Health shock on family member | 0.001 | -0.002 | 0.002 | 0.001 | 0.004 |
|  | (0.002) | (0.003) | (0.004) | (0.006) | (0.013) |
| Individual controls | Yes | Yes | Yes | Yes | Yes |
| Household controls | Yes | Yes | Yes | Yes | Yes |
| Province FE | Yes | Yes | Yes | Yes | Yes |
| Observations | 7341 | 3891 | 5221 | 5298 | 4825 |
| R-squared | 0.020 | 0.030 | 0.033 | 0.051 | 0.073 |

Notes: Standard errors are presented in parentheses. Individual control and family variables include gender, age, education level, marriage status, urban/rural status, income per family member, family size, and so forth. *** p<0.01, ** p<0.05, * p<0.1.

Table A7: Effects of Hospitalization and Insurance on Holding Bond or Financial Derivatives or Financial Instruments

|  | Holding Bond or Financial Derivatives or Financial Instrument | | | | |
| --- | --- | --- | --- | --- | --- |
|  | Bottom 20% | 20%-40% | 40%-60% | 60%-80% | Top 20% |
|  | (1) | (2) | (3) | (4) | (5) |
| Commercial medical insurance | 0.027** | 0.031 | 0.029** | 0.012 | 0.050*** |
|  | (0.014) | (0.019) | (0.014) | (0.014) | (0.019) |
| Social medical insurance | 0.002 | -0.003 | 0.004 | -0.003 | 0.069*** |
|  | (0.002) | (0.007) | (0.007) | (0.010) | (0.016) |
| Health shock | 0.000 | 0.004 | -0.004 | 0.009 | 0.005 |
|  | (0.002) | (0.004) | (0.004) | (0.008) | (0.016) |
| Health shock on family member | 0.002 | 0.002 | 0.004 | 0.006 | -0.006 |
|  | (0.002) | (0.004) | (0.005) | (0.007) | (0.015) |
| Individual controls | Yes | Yes | Yes | Yes | Yes |
| Household controls | Yes | Yes | Yes | Yes | Yes |
| Province FE | Yes | Yes | Yes | Yes | Yes |
| Observations | 7341 | 3891 | 5221 | 5298 | 4825 |
| R-squared | 0.024 | 0.042 | 0.043 | 0.043 | 0.091 |

Notes: Standard errors are presented in parentheses. Individual control and family variables include gender, age, education level, marriage status, urban/rural status, income per family member, family size, and so forth. *** p<0.01, ** p<0.05, * p<0.1.

Table A8: Financial Asset Allocation and Different Types of Insurance

|  | Upward Mobility | | | | Transition Probability | | | | Holding Risky Financial Asset | Proportion of Risky Financial Asset |
| --- | --- | --- | --- | --- | --- | --- | --- | --- | --- | --- |
|  | Bottom 20% | 20%-40% | 40%-60% | 60%-80% | Threshold: 20% | Threshold: 40% | Threshold: 60% | Threshold: 80% |  |  |
|  | (1) | (2) | (3) | (4) | (5) | (6) | (7) | (8) | (9) | (10) |
| Commercial medical insurance | 0.056* | 0.093** | 0.045 | -0.005 | 0.056* | 0.106*** | 0.088*** | 0.018 | 0.068*** | 0.036*** |
|  | (0.033) | (0.044) | (0.033) | (0.026) | (0.033) | (0.029) | (0.021) | (0.014) | (0.011) | (0.007) |
| Social medical insurance | 0.084*** | 0.103*** | 0.010 | 0.009 | 0.084*** | 0.067*** | 0.031*** | 0.012* | 0.012** | 0.005 |
|  | (0.020) | (0.031) | (0.028) | (0.023) | (0.020) | (0.015) | (0.011) | (0.007) | (0.005) | (0.003) |
| Commercial life insurance | 0.065* | 0.029 | 0.011 | 0.072*** | 0.065* | 0.048 | 0.030 | 0.044*** | 0.092*** | 0.056*** |
|  | (0.038) | (0.049) | (0.036) | (0.027) | (0.038) | (0.032) | (0.023) | (0.014) | (0.012) | (0.008) |
| Social endowment insurance | 0.025* | 0.024 | 0.007 | 0.032** | 0.025* | 0.026** | 0.012 | 0.003 | 0.004 | -0.001 |
|  | (0.015) | (0.022) | (0.018) | (0.015) | (0.015) | (0.011) | (0.008) | (0.005) | (0.003) | (0.002) |
| Social unemployment insurance | 0.018 | -0.010 | 0.067** | 0.024 | 0.018 | 0.013 | 0.036** | 0.033*** | 0.067*** | 0.033*** |
|  | (0.027) | (0.035) | (0.027) | (0.021) | (0.027) | (0.024) | (0.017) | (0.011) | (0.009) | (0.006) |
| Health shock | -0.058*** | -0.018 | -0.054*** | -0.001 | -0.058*** | -0.044*** | -0.038*** | -0.010* | -0.000 | 0.000 |
|  | (0.015) | (0.021) | (0.018) | (0.016) | (0.015) | (0.011) | (0.008) | (0.005) | (0.004) | (0.003) |
| Health shock on family member | -0.023 | -0.054** | -0.004 | 0.011 | -0.023 | -0.041*** | -0.017** | 0.003 | -0.001 | -0.003 |
|  | (0.015) | (0.021) | (0.017) | (0.016) | (0.015) | (0.011) | (0.008) | (0.005) | (0.004) | (0.003) |
| Individual controls | Yes | Yes | Yes | Yes | Yes | Yes | Yes | Yes | Yes | Yes |
| Household controls | Yes | Yes | Yes | Yes | Yes | Yes | Yes | Yes | Yes | Yes |
| Province FE | Yes | Yes | Yes | Yes | Yes | Yes | Yes | Yes | Yes | Yes |
| Observations | 7341 | 3891 | 5221 | 5298 | 7341 | 11232 | 16453 | 21751 | 26576 | 26576 |
| R-squared | 0.146 | 0.121 | 0.120 | 0.134 | 0.146 | 0.155 | 0.149 | 0.132 | 0.202 | 0.160 |

Notes: Standard errors are presented in parentheses. Individual control and family variables include gender, age, education level, marriage status, urban/rural status, income per family member, family size, and so forth. *** p<0.01, ** p<0.05, * p<0.1.

Table A9: Medical Insurance Premium and Household Financial Asset Allocation

|  | Holding Risky Financial Asset | Proportion of Risky Financial Asset |
| --- | --- | --- |
|  | (1) | (2) |
| Log of Commercial Medical Insurance Premium | 0.013*** | 0.008*** |
|  | (0.002) | (0.001) |
| Log of Social Medical Insurance Premium | -0.002** | -0.002*** |
|  | (0.001) | (0.001) |
| Health shock | 0.001 | 0.001 |
|  | (0.004) | (0.003) |
| Health shock on family member | -0.004 | -0.004 |
|  | (0.004) | (0.002) |
| Individual controls | Yes | Yes |
| Household controls | Yes | Yes |
| Province FE | Yes | Yes |
| Observations | 24188 | 24188 |
| R-squared | 0.193 | 0.155 |

Notes: Standard errors are presented in parentheses. Individual control and family variables include gender, age, education level, marriage status, urban/rural status, income per family member, family size, and so forth. *** p<0.01, ** p<0.05, * p<0.1.

Table A10: Logit Models: Financial Asset Mobility and Allocation

|  | Upward Mobility | | | | Transition Probability | | | |
| --- | --- | --- | --- | --- | --- | --- | --- | --- |
|  | Bottom 20% | 20%-40% | 40%-60% | 60%-80% | Threshold: 20% | Threshold: 40% | Threshold: 60% | Threshold: 80% |
|  | (1) | (2) | (3) | (4) | (5) | (6) | (7) | (8) |
| Hospitalization | -0.162*** | -0.054 | -0.173*** | -0.015 | -0.162*** | -0.148*** | -0.175*** | -0.108*** |
|  | (0.042) | (0.060) | (0.059) | (0.063) | (0.042) | (0.036) | (0.035) | (0.039) |
| Hospitalization: family member | -0.064 | -0.148** | -0.012 | 0.039 | -0.064 | -0.123*** | -0.069** | 0.001 |
|  | (0.042) | (0.059) | (0.052) | (0.059) | (0.042) | (0.035) | (0.032) | (0.036) |
| Social medical insurance | 0.254*** | 0.315*** | 0.056 | 0.083 | 0.254*** | 0.262*** | 0.168*** | 0.136** |
|  | (0.058) | (0.090) | (0.084) | (0.098) | (0.058) | (0.053) | (0.050) | (0.059) |
| Commercial medical insurance | 0.267** | 0.306** | 0.133 | 0.072 | 0.267** | 0.351*** | 0.285*** | 0.134** |
|  | (0.121) | (0.125) | (0.087) | (0.085) | (0.121) | (0.083) | (0.061) | (0.057) |
| Individual controls | Yes | Yes | Yes | Yes | Yes | Yes | Yes | Yes |
| Household controls | Yes | Yes | Yes | Yes | Yes | Yes | Yes | Yes |
| Province FE | Yes | Yes | Yes | Yes | Yes | Yes | Yes | Yes |
| Observations | 7340 | 3888 | 5215 | 5295 | 7340 | 11225 | 16447 | 21740 |
| Pseudo R-squared | 0.119 | 0.092 | 0.096 | 0.124 | 0.119 | 0.126 | 0.141 | 0.181 |

Notes: Standard errors are presented in parentheses. Individual control and family variables include gender, age, education level, marriage status, urban/rural status, income per family member, family size, and so forth. *** p<0.01, ** p<0.05, * p<0.1.

Table A11: Effects of Decline in Self-reported Health Status and Insurance on Proportion of Risky Financial Asset

|  | Proportion of Risky Financial Asset | | | | |
| --- | --- | --- | --- | --- | --- |
|  | Bottom 20% | 20%-40% | 40%-60% | 60%-80% | Top 20% |
|  | (1) | (2) | (3) | (4) | (5) |
| Commercial medical insurance | 0.015 | 0.039** | 0.030** | 0.038*** | 0.066*** |
|  | (0.011) | (0.017) | (0.012) | (0.014) | (0.015) |
| Social medical insurance | 0.003 | -0.003 | 0.010* | -0.017 | 0.043*** |
|  | (0.002) | (0.007) | (0.005) | (0.011) | (0.015) |
| Decline in Self-reported Health Status | -0.003* | -0.001 | -0.003 | -0.002 | -0.011 |
|  | (0.001) | (0.003) | (0.003) | (0.005) | (0.009) |
| Individual controls | Yes | Yes | Yes | Yes | Yes |
| Household controls | Yes | Yes | Yes | Yes | Yes |
| Province FE | Yes | Yes | Yes | Yes | Yes |
| Observations | 7344 | 3895 | 5225 | 5302 | 4828 |
| R-squared | 0.043 | 0.055 | 0.080 | 0.083 | 0.146 |

Notes: Standard errors are presented in parentheses. Individual control and family variables include gender, age, education level, marriage status, urban/rural status, income per family member, family size, and so forth. *** p<0.01, ** p<0.05, * p<0.1.

Table A12: Effects of Decline in Self-reported Health Status and Insurance on Holding Risky Financial Asset

|  | Holding Risky Financial Asset | | | | |
| --- | --- | --- | --- | --- | --- |
|  | Bottom 20% | 20%-40% | 40%-60% | 60%-80% | Top 20% |
|  | (1) | (2) | (3) | (4) | (5) |
| Commercial medical insurance | 0.022 | 0.071*** | 0.073*** | 0.077*** | 0.103*** |
|  | (0.017) | (0.027) | (0.021) | (0.022) | (0.022) |
| Social medical insurance | 0.002 | 0.003 | 0.021** | -0.015 | 0.084*** |
|  | (0.004) | (0.009) | (0.010) | (0.016) | (0.024) |
| Decline in Self-reported Health Status | -0.002 | -0.003 | -0.006 | -0.004 | -0.023 |
|  | (0.003) | (0.005) | (0.006) | (0.008) | (0.014) |
| Individual controls | Yes | Yes | Yes | Yes | Yes |
| Household controls | Yes | Yes | Yes | Yes | Yes |
| Province FE | Yes | Yes | Yes | Yes | Yes |
| Observations | 7344 | 3895 | 5225 | 5302 | 4828 |
| R-squared | 0.055 | 0.065 | 0.094 | 0.107 | 0.176 |

Notes: Standard errors are presented in parentheses. Individual control and family variables include gender, age, education level, marriage status, urban/rural status, income per family member, family size, and so forth. *** p<0.01, ** p<0.05, * p<0.1.

Table A13: Effects of Decline in Self-reported Health Status and Insurance on Transition Probability

|  | Transition Probability | | | |
| --- | --- | --- | --- | --- |
|  | Threshold: 20% | Threshold: 40% | Threshold: 60% | Threshold: 80% |
|  | (1) | (2) | (3) | (4) |
| Commercial medical insurance | 0.075** | 0.120*** | 0.096*** | 0.031** |
|  | (0.032) | (0.028) | (0.020) | (0.013) |
| Social medical insurance | 0.086*** | 0.073*** | 0.035*** | 0.014** |
|  | (0.020) | (0.015) | (0.011) | (0.007) |
| Decline in Self-reported Health Status | -0.031*** | -0.025*** | -0.016** | -0.004 |
|  | (0.012) | (0.009) | (0.006) | (0.004) |
| Individual controls | Yes | Yes | Yes | Yes |
| Household controls | Yes | Yes | Yes | Yes |
| Province FE | Yes | Yes | Yes | Yes |
| Observations | 7344 | 11239 | 16464 | 21766 |
| R-squared | 0.144 | 0.153 | 0.147 | 0.131 |

Notes: Standard errors are presented in parentheses. Individual control and family variables include gender, age, education level, marriage status, urban/rural status, income per family member, family size, and so forth. *** p<0.01, ** p<0.05, * p<0.1.

Table A14: Effects of Decline in Self-reported Health Status and Insurance on Upward mobility

|  | Upward mobility | | | |
| --- | --- | --- | --- | --- |
|  | Bottom 20% | 20%-40% | 40%-60% | 60%-80% |
|  | (1) | (2) | (3) | (4) |
| Commercial medical insurance | 0.075** | 0.101** | 0.049 | 0.017 |
|  | (0.032) | (0.040) | (0.031) | (0.025) |
| Social medical insurance | 0.086*** | 0.111*** | 0.018 | 0.020 |
|  | (0.020) | (0.030) | (0.027) | (0.023) |
| Decline in Self-reported Health Status | -0.031*** | -0.028* | -0.022 | 0.000 |
|  | (0.012) | (0.017) | (0.014) | (0.012) |
| Individual controls | Yes | Yes | Yes | Yes |
| Household controls | Yes | Yes | Yes | Yes |
| Province FE | Yes | Yes | Yes | Yes |
| Observations | 7344 | 3895 | 5225 | 5302 |
| R-squared | 0.144 | 0.119 | 0.117 | 0.131 |

Notes: Standard errors are presented in parentheses. Individual control and family variables include gender, age, education level, marriage status, urban/rural status, income per family member, family size, and so forth. *** p<0.01, ** p<0.05, * p<0.1.

Table A15: IV regressions - Effects of Hospitalization and Insurance on Transition Probability

|  | Transition Probability | | | |
| --- | --- | --- | --- | --- |
|  | Threshold: 20% | Threshold: 40% | Threshold: 60% | Threshold: 80% |
|  | (1) | (2) | (3) | (4) |
| Commercial medical insurance | 0.304* | 0.317*** | 0.233*** | 0.031 |
|  | (0.181) | (0.114) | (0.066) | (0.035) |
| Social medical insurance | 0.094*** | 0.079*** | 0.040*** | 0.015* |
|  | (0.020) | (0.016) | (0.012) | (0.008) |
| Health shock | -0.058*** | -0.045*** | -0.039*** | -0.010* |
|  | (0.014) | (0.011) | (0.008) | (0.006) |
| Health shock on family member | -0.022 | -0.038*** | -0.016** | 0.003 |
|  | (0.015) | (0.011) | (0.008) | (0.005) |
| Individual controls | Yes | Yes | Yes | Yes |
| Household controls | Yes | Yes | Yes | Yes |
| Province FE | Yes | Yes | Yes | Yes |
| First-stage F statistics | 12.68 | 21.81 | 36.13 | 56.58 |
| Observations | 7341 | 11232 | 16453 | 21751 |
| R-squared | 0.140 | 0.150 | 0.145 | 0.131 |

Notes: Individual control and family variables include gender, age, education level, marriage status, urban/rural status, income per family member, family size, and so forth. *** p<0.01, ** p<0.05, * p<0.1. First-stage F statistics are reported in the table.

Table A16: IV regressions - Effects of Hospitalization and Insurance on Upward Mobility

|  | Upward mobility | | | |
| --- | --- | --- | --- | --- |
|  | Bottom 20% | 20%-40% | 40%-60% | 60%-80% |
|  | (1) | (2) | (3) | (4) |
| Commercial medical insurance | 0.304* | 0.442*** | 0.091 | -0.065 |
|  | (0.181) | (0.166) | (0.103) | (0.073) |
| Social medical insurance | 0.094*** | 0.120*** | 0.020 | 0.017 |
|  | (0.020) | (0.032) | (0.027) | (0.024) |
| Health shock | -0.058*** | -0.022 | -0.054*** | -0.001 |
|  | (0.014) | (0.022) | (0.019) | (0.016) |
| Health shock on family member | -0.022 | -0.048** | -0.005 | 0.012 |
|  | (0.015) | (0.021) | (0.018) | (0.016) |
| Individual controls | Yes | Yes | Yes | Yes |
| Household controls | Yes | Yes | Yes | Yes |
| Province FE | Yes | Yes | Yes | Yes |
| First-stage F statistics | 12.68 | 10.38 | 14.96 | 19.74 |
| Observations | 7341 | 3891 | 5221 | 5298 |
| R-squared | 0.140 | 0.106 | 0.118 | 0.129 |

Notes: Individual control and family variables include gender, age, education level, marriage status, urban/rural status, income per family member, family size, and so forth. *** p<0.01, ** p<0.05, * p<0.1. First-stage F statistics are reported in the table.

Table A17: IV regressions - Effects of Hospitalization and Insurance on Holding Risky Financial Asset

|  | Holding Risky Financial Asset | | | | |
| --- | --- | --- | --- | --- | --- |
|  | Bottom 20% | 20%-40% | 40%-60% | 60%-80% | Top 20% |
|  | (1) | (2) | (3) | (4) | (5) |
| Commercial medical insurance | 0.023 | 0.071*** | 0.073*** | 0.077*** | 0.103*** |
|  | (0.017) | (0.027) | (0.020) | (0.022) | (0.022) |
| Social medical insurance | 0.002 | 0.002 | 0.021** | -0.016 | 0.085*** |
|  | (0.004) | (0.009) | (0.010) | (0.016) | (0.024) |
| Health shock | -0.001 | 0.003 | -0.010 | 0.023* | -0.001 |
|  | (0.003) | (0.006) | (0.007) | (0.012) | (0.019) |
| Health shock on family member | 0.006 | -0.004 | 0.012 | 0.004 | -0.032* |
|  | (0.004) | (0.005) | (0.008) | (0.011) | (0.018) |
| Individual controls | Yes | Yes | Yes | Yes | Yes |
| Household controls | Yes | Yes | Yes | Yes | Yes |
| Province FE | Yes | Yes | Yes | Yes | Yes |
| First-stage F statistics | 12.68 | 10.38 | 14.96 | 19.74 | 18.63 |
| Observations | 7341 | 3891 | 5221 | 5298 | 4825 |
| R-squared | 0.055 | 0.065 | 0.095 | 0.107 | 0.176 |

Notes: Individual control and family variables include gender, age, education level, marriage status, urban/rural status, income per family member, family size, and so forth. *** p<0.01, ** p<0.05, * p<0.1. First-stage F statistics are reported in the table.

Table A18: IV regressions - Effects of Hospitalization and Insurance on Proportion of Risky Financial Asset

|  | Proportion of Risky Financial Asset | | | | |
| --- | --- | --- | --- | --- | --- |
|  | Bottom 20% | 20%-40% | 40%-60% | 60%-80% | Top 20% |
|  | (1) | (2) | (3) | (4) | (5) |
| Commercial medical insurance | 0.016 | 0.039** | 0.030** | 0.038*** | 0.066*** |
|  | (0.011) | (0.017) | (0.012) | (0.014) | (0.015) |
| Social medical insurance | 0.003 | -0.003 | 0.011* | -0.018* | 0.044*** |
|  | (0.002) | (0.007) | (0.005) | (0.011) | (0.015) |
| Health shock | -0.001 | 0.000 | -0.005 | 0.019** | -0.001 |
|  | (0.002) | (0.003) | (0.004) | (0.008) | (0.013) |
| Health shock on family member | 0.002 | -0.001 | 0.002 | -0.000 | -0.022* |
|  | (0.002) | (0.003) | (0.004) | (0.006) | (0.011) |
| Individual controls | Yes | Yes | Yes | Yes | Yes |
| Household controls | Yes | Yes | Yes | Yes | Yes |
| Province FE | Yes | Yes | Yes | Yes | Yes |
| First-stage F statistics | 12.68 | 10.38 | 14.96 | 19.74 | 18.63 |
| Observations | 7341 | 3891 | 5221 | 5298 | 4825 |
| R-squared | 0.043 | 0.055 | 0.080 | 0.084 | 0.146 |

Notes: Individual control and family variables include gender, age, education level, marriage status, urban/rural status, income per family member, family size, and so forth. *** p<0.01, ** p<0.05, * p<0.1. First-stage F statistics are reported in the table.

**Figure A1:** Propensity-score-matching result

**Figure A2:** Self-paid premium for social medical insurance


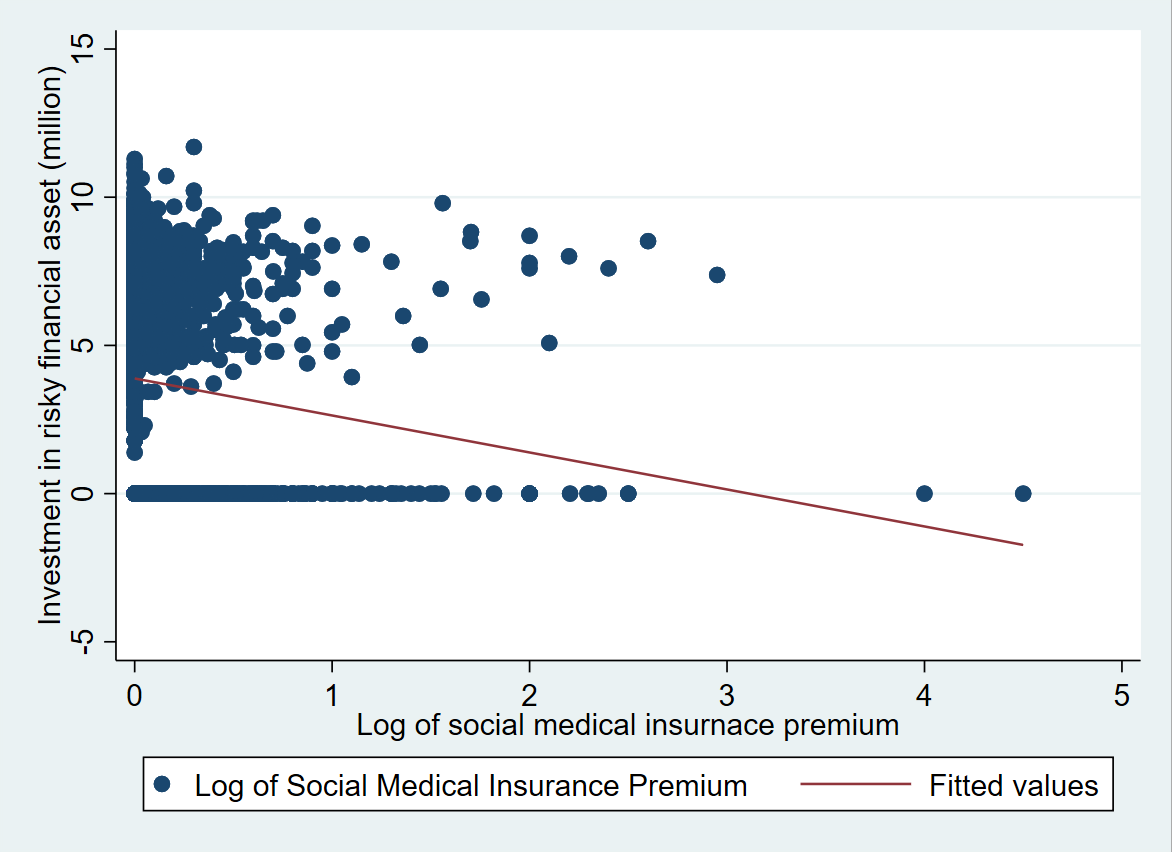


**Figure A3:** Correlation between social medical insurance premium and risky financial asset investment
